# Simultaneous identification of long similar substrings in large sets of sequences

**DOI:** 10.1186/1471-2105-8-S5-S7

**Published:** 2007-05-24

**Authors:** Jürgen Kleffe, Friedrich Möller, Burghardt Wittig

**Affiliations:** 1 Institut für Molekularbiologie und Bioinformatik, Charite-Universitätsmedizin Berlin, Arnimallee 22, 14195 Berlin, Germany

## Abstract

**Background:**

Sequence comparison faces new challenges today, with many complete genomes and large libraries of transcripts known. Gene annotation pipelines match these sequences in order to identify genes and their alternative splice forms. However, the software currently available cannot simultaneously compare sets of sequences as large as necessary especially if errors must be considered.

**Results:**

We therefore present a new algorithm for the identification of almost perfectly matching substrings in very large sets of sequences. Its implementation, called ClustDB, is considerably faster and can handle 16 times more data than VMATCH, the most memory efficient exact program known today. ClustDB simultaneously generates large sets of exactly matching substrings of a given minimum length as seeds for a novel method of match extension with errors. It generates alignments of maximum length with a considered maximum number of errors within each overlapping window of a given size. Such alignments are not optimal in the usual sense but faster to calculate and often more appropriate than traditional alignments for genomic sequence comparisons, EST and full-length cDNA matching, and genomic sequence assembly. The method is used to check the overlaps and to reveal possible assembly errors for 1377 *Medicago truncatula *BAC-size sequences published at .

**Conclusion:**

The program ClustDB proves that window alignment is an efficient way to find long sequence sections of homogenous alignment quality, as expected in case of random errors, and to detect systematic errors resulting from sequence contaminations. Such inserts are systematically overlooked in long alignments controlled by only tuning penalties for mismatches and gaps.

ClustDB is freely available for academic use.

## Background

More than 500 eukaryotic genome projects are on the way and will sooner or later generate hundreds of billion base pairs. The accurate annotation of genes in these many sequences is a challenging computational problem and impossible to complete by experimental methods alone. However, it is also estimated that about 50% of an organism's genes can be identified by strong sequence similarity to other organisms. The resulting sequence matching problems require simultaneous comparisons of large sets of sequences and are no longer efficiently handled by matching in turn each candidate sequence after the other as does BLAST and its various improvements including BLAT [1]. Using suffix trees and suffix arrays, more efficient and exact methods of simultaneous sequence comparison exist. These methods quickly identify perfect matches of substrings. Their application is justified by the observation, that approximately identical sequences have exact common substrings which are often specific enough to identify sequence similarities of interest. Errors are rarely randomly distributed but cluster, leaving space for longer exact matches than expected from probability computations. Hence, by almost perfect sequence matching, we can easily locate BACs and shorter sequences on chromosomes, relate single ESTs to full-length cDNA and identify redundant and contaminated sequences. For instance, such methods quickly reveal large numbers of almost identical human ESTs stored in Genbank which cause largely increased multiple output of spliced alignment programs, genome browsers use to map ESTs on chromosomes. One of the largest subset counts 237 human ESTs which are not human transcripts but part of the *E. coli *vector AF058756 (Cloning vector pFR-Luc) used for sequence amplification. Sorek and Safer [2] describe various kinds of EST contamination which are all detectable by analyzing matching substrings. By statistical analysis of complete EST libraries these authors found 24,766 human ESTs which are likely contaminated by intergenic, intronic or repetitive DNA, and may have caused up to 9,575 incorrect gene predictions. By exact string matching Kurtz et al. [3] discovered a 190,014 bp repeat in the human chromosome 22 contig 8 caused by wrong sequence assembly that has been corrected. The Genbank sequence of *Arabidopsis thaliana *chromosome III still contains a 5,452 base pairs fraction of the cloning vector pBACe3.6 at position 13,754,155. Four overlapping repeats found in chromosome IV are caused by a nine-fold tandem repeat of 3,259 base pairs that needs careful investigation. Such facts are generally discovered by chance since existing methods for sequence matching cannot simultaneously compare sequence data as large as necessary. The suffix tree approach requires too much memory. Although the program MUMMER by Delcher et al. [4] is based on a more space efficient suffix tree implementation which is also used in REPFIND by Kurtz and Schleiermacher [5], it cannot deal with more than 120 MB of sequence at a time using even 2 GB of RAM. The next better program VMATCH by Abouelhoda et al. [6] uses an enhanced suffix array. Its recent version can handle at most 250 MB of sequence using the same amount of memory. Other methods by Burkhardt et al. [7], Höhl et al. [8] and Lefebvre et al [9], also require at least 8 bytes of memory for each base pair to compare. However, we will soon need programs which are able to compare more base pairs of sequence than there are bytes of RAM. One such program is ClustDB [10] based on a partitioned suffix array method. It needs less than four hours to simultaneously compare 3.3 GB of human ESTs using a PC with 2 GB of RAM. This program was further improved by a novel algorithm for match extension with errors which is the subject of this paper.

## Results and discussion

### Quality of window-alignment

**Figure 1:**
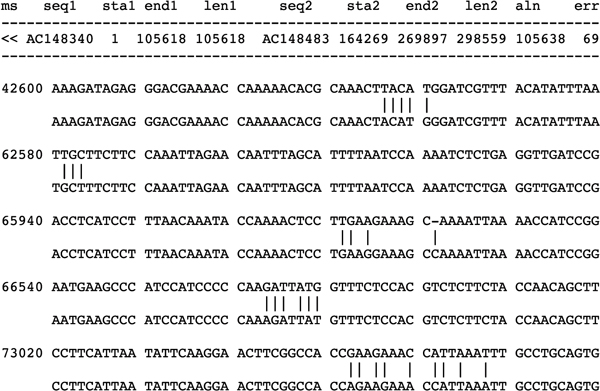
**Alignment output of ClustDB**. The BAC AC148340 (len1 = 105618 bp) is approximately contained in the BAC AC148483 (len2 = 298559 bp). The alignment has length aln = 105638 including 20 gaps in AC148340 and 9 gaps in AC148483. Its displayed part shows five clusters of mismatches surrounded by long perfect matches so that the number of errors does not exceed 10 in each window of size 40. Therefore, ClustDB does not look for improvements by introducing gaps and reports 69 errors, i.e. 16 errors more than the exact alignment computes. Note that each cluster of mismatches can be realigned with two gaps.

ClustDB extends to both sides all left maximal pairs of substrings exactly matching over at least *M *characters until a given number of errors *K *is exceeded in a window of given size *W*. This approach controls match quality independent of alignment length. Its complete match option confines on listing sequences contained in others and pairs of sequences which overlap. The total number of errors could be controlled, too, as implemented in VMATCH [6]. But this approach turned out to be impractical for large sets of sequences. From one exact match it often generates large numbers of differently extended matches while our approach reduces output by generating identical match extensions from different exact matches. We applied ClustDB to a set of 2020 *Medicago truncatula *BACs published at the website  and found 1215 complete matches using the parameters *M *= 100, *W *= 40 and *K *= 10. The numbers of errors reported were compared with the optimal alignment scores which took more than 30 hours to calculate even knowing the start and end positions of all matches. Only 64 out of 1215 cases showed differences to the optimal alignment score in either the number of mismatches or gaps. The total number of errors differed for 57 cases for which a histogram is shown in Table 1. The largest difference was 16 errors over an alignment of more than 100 kbp, a case discussed in the legend of Figure 1.

### ClustDB outperforms VMATCH in speed and sensitivity

We applied ClustDB to find redundant sequences in a smaller set of 997 finished *Medicago *BACs downloaded from the NCBI (September 15th, 2005) in order to identify overlapping sequences and sequences which are contained in others. Consideration of both strands yields 1994 sequences which add up to about 220 MB. Using *M *= 100, *W *= 20 and *K *= 5, ClustDB takes 11:37 minutes to identify 510 complete sequence matches, i.e. those which identify overlapping BACs and those contained in others. Much shorter run times are obtained for larger values of *M*. For *M *= 1000 ClustDB takes only 6:34 minutes to derive 465 complete matches admitting 5 errors in each window of size 20. Hence, 45 complete matches do not include an exact match of length 1000. The calculations were performed on a Pentium 4 PC with 2 GB of RAM and 2.6 GHz processor speed.

On the same computer, VMATCH takes 16:40 minutes to find only 116 exact complete matches for *M *= 100 or 16:10 minutes to find only 66 exact complete matches with *M *= 1000. In this program *M *is called seed string length. Admitting at most 10 errors – the maximal error count allowed in VMATCH – it takes 17:08 minutes to derive 348 complete matches for *M *= 100 and 16:23 minutes to find 236 complete matches for *M *= 1000. Furthermore the two sets of complete matches derived by ClustDB contain 414 and 381 complete matches with at most 10 errors. VMATCH should find all of these matches but fails. For technical reasons it considers the nucleotide 'n' to mismatch all other nucleotides including itself while ClustDB takes the biologically more correct point of view and considers the letter 'n' to match all other nucleotides and finds more matches this way. Hence, ClustDB outperforms VMATCH in speed and sensitivity.

Runtime basically depends on how many matches are found. For another larger and match rich sample of 1377 BACs as well as their complementary sequences (324 MB) described in the next section ClustDB took considerably longer times of execution shown in Table 2. The shortest time is 25 minutes for *M *= 100 *W *= 50 and *K *= 5. VMATCH performed very differently on this data set. While it was fast in generating all extended matches with at most 6 errors and seed string length *M *= 100 (27 minutes) it suddenly took more than 20 hours to derive all matches with up to 7 and more errors. The reason could be a bug in the program or an exponential increase of match extensions found. VMATCH extends every left maximal pair of exactly matching substrings while ClustDB does not extend matches which are contained in an already extended match. It is also shown in [10] that VMATCH is on its limit by processing about 220 MB of sequence with 2 GB of RAM while the latest version of ClustDB handles up to 4 GB of sequences. Hence, swapping may be a reason, too. Moreover, the output of VMATCH makes not much sense for such problems and requests a massive additional effort to derive the results obtained by ClustDB using the output of VMATCH.

### Testing ClustDB on TIGR's current Medicago BAC assembly

The website  offers 9 BAC assembly files named contig_lg0.dat to contig_lg8.dat. They contain 407 chains of sequences claimed to overlap by more than 1000 nucleotides for each junction. The total number of sequences in these chains is 1377 and they contain 969 overlapping sequence pairs which form our test set for ClustDB. Of these 969 sequence pairs 799 (82%) were confirmed by application of ClustDB to the 1377 BACs and their complementary sequences (324 MB) using parameters *M *= 100, *W *= 100, *K *= 30, the complete match option, and reporting only extended matches of length greater than 1000. Interestingly, these are in fact all correctly published overlaps of complete sequences as our following study shows.

ClustDB was applied to the remaining 170 pairs with the complete match option switched off. The overlapping property could not even partly be confirmed for five pairs CT971491/CT961056, AC171618*/AC160842*, AC147960/CR931732, AC153162*/AC159662* and AC158173*/AC140022. The asterisk denotes working draft sequences which are incomplete and consist of a number of unordered sequence sections which must be considered separate sequences for a meaningful application of ClustDB. TIGR claims no confidence in the results published for these sequences. The two pairs of complete sequences CT971491/CT961056 and AC147960/CR931732 were studied using BLAST and no significant local alignments longer than 300 bp were found. Trusting BLAST, we are convinced that these two cases are no real overlaps. In another 12 cases no match extended until one of both sequence ends. But in all these cases at least one and mostly both sequences were working draft sequences or had the tag "sequencing in progress" in their GenBank LOCUS fields. For the same reason we did not consider another 133 pairs of BACs.

**Figure 2:**
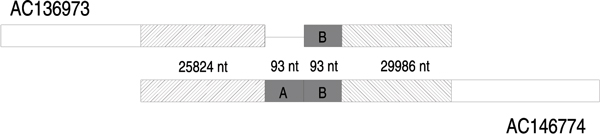
**Window Alignment detects sequence contamination**. The BAC AC146774 is possibly contaminated by an approximate copy A (93 nt) of a low complexity subsequence section B. Removing A from AC146774 yields a perfect alignment with one mismatch.

The remaining 20 pairs of complete sequences are listed in Table 3. Figure 2 shows the kind of match found in 16 of these cases (type 1). Two excellent matches extend from both sides of the overlap, but a poorly matching sequence section stops the window alignment in nearby sequence positions. Note that if the match extensions are not complete there are necessarily *K *+ 1 = 31 errors in the last window of size *W *= 100. Hence, if the total number of errors is 32, only one error occurs in the remaining part of the match indicating a very inhomogeneous alignment quality.

In three other cases (type 2), ClustDB detects one match reaching from the start of the overlap to shortly before its end missing 236, 414 and 409 nucleotides, respectively. Again, almost all errors occur in the last alignment window and hence, the tails of the upstream BACs should be studied. The overlap claimed by TIGR for the sequence pair AC142394/AC147435 splits into two sections (type 3). One section of length 15702 aligns badly with 7893 errors (about 50%) and is followed by a section of length 85901 that aligns perfectly with only 5 errors. This places serious doubts on the correctness of both BACs.

### Alternative assembly

**Figure 3:**
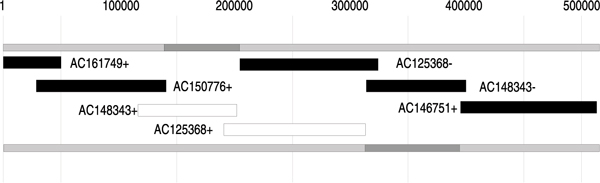
**Window Alignment detects alternative assemblies**. Displayed is a part of the temporary BAC assembly by the *Medicago *sequencing consortium drawn to scale using + and - signs to indicate sequence strand. Five BACs (black boxes) are arranged into two assemblies separated by a 60 kbp gap of unknown sequence. However, a long exact match of AC148343+ with AC150776+ suggests another possible assembly indicated by white boxes. See Table 4 for the match quality of all considered overlaps.

ClustDB applied to a large set of BACs, simultaneously, does not only confirm known matches but also detects large numbers of new alternative matches which are worth to consider. Our application described in the previous section produced 1015 complete matches of which at least 36 are not listed in the published *Medicago *BAC assembly tables which suggest alternative BAC assemblies. Just one example is discussed in Figure 3. It compares a BAC assembly derived from our complete matches (white boxes) with the result provided by the *Medicago *sequencing consortium (black boxes). Table 4 presents the match errors for all involved pairs of overlapping BACs. There is a 21493 bp long exact overlap of sequence AC150776+ with the sequence AC148343+ (white box). It suggests an alternative assembly that covers the about 60 kbp gap of unknown sequence. However, there is no proof of it. Assuming the consortium's assembly is correct, a long inverted repeat exists as has been observed very frequently in the human X chromosome [11]. We are far from solving this puzzle here, but this case proves the importance of good methods for sequence comparisons that make us think about such problems.

## Conclusion

We proved window alignment an efficient way to find long sections of similar sequences. Compared with traditional alignment, it is faster to calculate, reveals sequence sections of homogenous alignment quality, just such as expected for random errors, and also detects local systematic errors like sequence contaminations. Such inserts are often overlooked by optimizing global scores for long alignments only tuning parameters like penalties for mismatches and gaps. The case depicted in Figure 2 has an excellent global alignment score and still shows a severe local sequence matching problem that is important to detect. However, not only the quality of individual alignments but also the speed of simultaneous comparison of large set of sequences makes ClustDB an indispensable tool for genomic sequence analysis.

We showed that ClustDB outperforms VMATCH in comparing BAC size sequences mainly caused by an inappropriate match extension method and the high memory consumption of the latter program. Application of the software BLAT to our data was stopped after several days of endless computation. Note that BAC assembly is just one application of ClustDB. Our alignment concept is also beneficial for mRNA to cDNA comparison in order to confirm exons, introns and alternative splice sites. Alignments used in such contexts should tolerate sufficiently spaced single errors in larger numbers than dense blocks of errors. The program GenomeFlicer by Mielordt et al. [12] admits at most 2 errors in a window of length 20 in order to confirm genes and to distinguish NAGNAG acceptor isoforms [13]. We also expect that ClustDB helps to study highly repetitive genes, a problem described especially challenging by Check [14]. We developed ClustDB to play a major role in future genome wide comparisons of genes and currently work on specialized program versions for EST- and full-length cDNA matching, genome wide detection of alternative splicing as well as distributed parallel processing [15].

## Methods

### Algorithm

The key concept used in ClustDB [10] is a linear space representation of all left maximal sets of common substrings of a given length *M*, called substring-clusters. A cluster of identical substrings is called left maximal if at least two identical substrings in this cluster are preceded by different letters. Other clusters of common substrings need not be considered for sequence matching. The members of each cluster are given by sets of triplets (*c*, *s*, *p*) with the same value of *c*, called cluster number, *s *denotes sequence number and *p *denotes start position of the substring within sequence *s*. These triplets are stored in linear space and are quickly derived in approximately linear time based on a novel count sort method performed on the place. Next they are used to efficiently list pairs of left maximal matching substrings, and to simultaneously extend such pairs to maximum length as shown in [10]. Let us here describe how ClustDB further extends such pairs to both sides until a given number of errors *K *is exceeded in a window of given size *W*. This window approach easily detects similar sequences, sequences contained in others and pairs of sequences which overlap.

In the program ClustDB all string comparison is done using a long sequence formed by concatenation of all individual sequences separated by dot characters used as end-of-sequence symbols. Hence, pairs of matching substrings are internally represented by triplets (*p1*, *p2*, *n*) where *p1 *<*p2 *are the start positions in the concatenated sequence and *n *is the length of the match. In order to avoid unnecessary match extensions with mismatches, we alternatively present the maximally extended exact matches by triplets (*p*, *o*, *n*) with *p *= *p1 *and offset *o *= *p2 *- *p1 *> 0. Then, after grouping all triplets with the same offset and sorting each group in increasing order of *p*, a maximally extended match allowing mismatches but no gaps is prone to contain the next not yet extended exact match that needs not be considered. The result is a shorter list of maximally extended pairwise matches with mismatches represented by quadruplets (*p*, *o*, *n*, *e*). The new integer *e *is the number of mismatches and forms an upper bound for the edit distance. Such extended match has at most *K *mismatches within each internal window of size *W*. The two boundary windows have *K *+ 1 errors or reach end-of-sequence symbols.

For match extension with gaps, we construct an as long as possible virtual alignment that contains the considered match and has the property that each internal alignment window of size *W *has at most *K *errors using edit distance. This alignment is never stored during computation. Instead we only store and calculate the vectors (*p1*, *e1*, *p2*, *e2*, *e*, *a*) where *p1*, *e1 *and *p2*, *e2 *are the start and end positions of the extended match. The integer *a *stands for the length of the virtual alignment including gap symbols and is necessary for the calculation of the numbers of gaps *g1 *= *a *- *e1 *+ *p1 *- 1 in substring 1 and *g2 *= *a *- *e2 *+ *p2 *- 1 in substring 2, respectively. The number of mismatches follows from *m *= *e *- *g1 *- *g2*. On request, complete printouts of alignments are generated from stored lists of gap positions relative to *p1 *and *p2*. Each match is first extended to the left, than to the right which we will describe in more detail.

**Figure 4:**
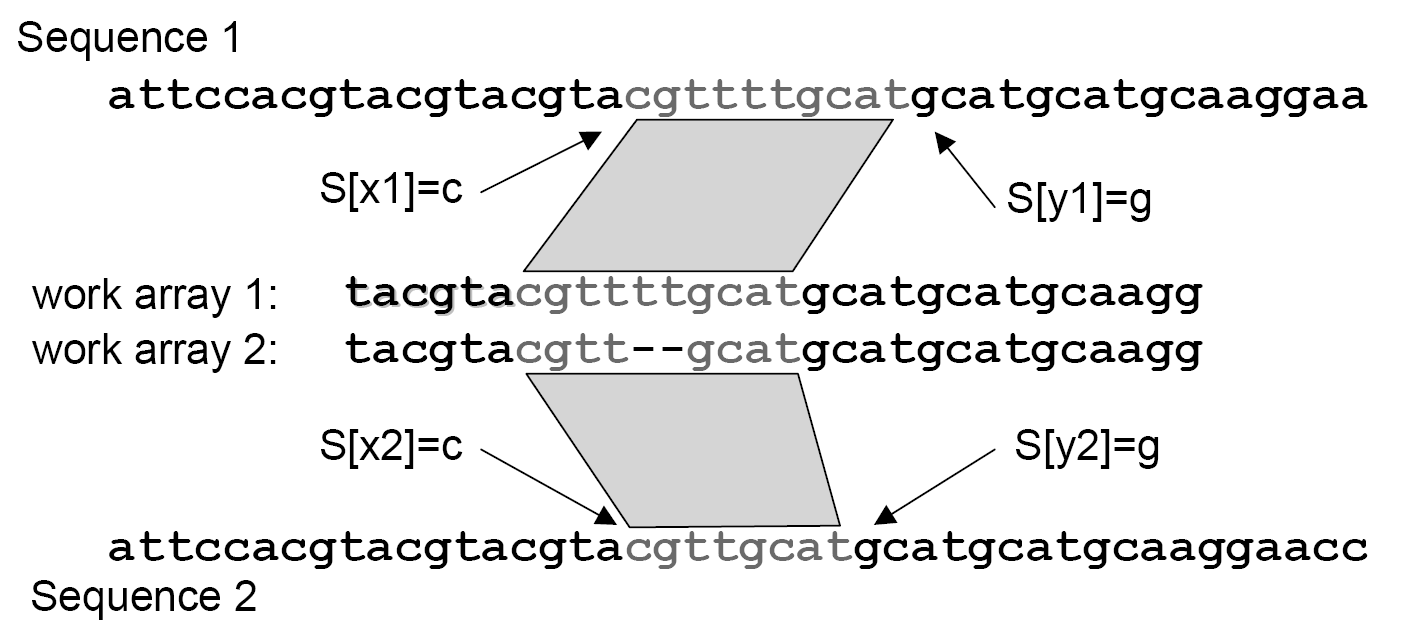
**The principle of window alignment**. Definition of the variables *x1*, *y1 *and *x2*, *y2 *used for match extension with gaps. Sequence 1 and Sequence 2 are matching parts of the concatenated long sequences S that is stored in memory. The sliding window of length 10 is marked by grey colour.

Assume a match is maximally extended to the right allowing no gaps and at most *K *mismatches in each internal window of size *W*. The boundary window to the right has *K *+ 1 errors or reaches the end of at least one sequence. Nothing remains to do in the latter case. Otherwise, we aim to reduce the number of errors of the rightmost window by calculating an alignment with gaps. For this reason, we use two work arrays of size 3*W *in order to calculate and evaluate optimal alignments by moving a window of size *W *along the aligned sequences stored in the two work arrays. Two pairs of variables (*x1*, *y1*) and (*x2*, *y2*) link the four corners of the sliding window with the real positions in the sequences as seen in Figure 4. Note that *y1 *- *x1 *and *y2 *- *x2 *may be less than *W *if the sliding window includes gaps.

**Figure 5:**
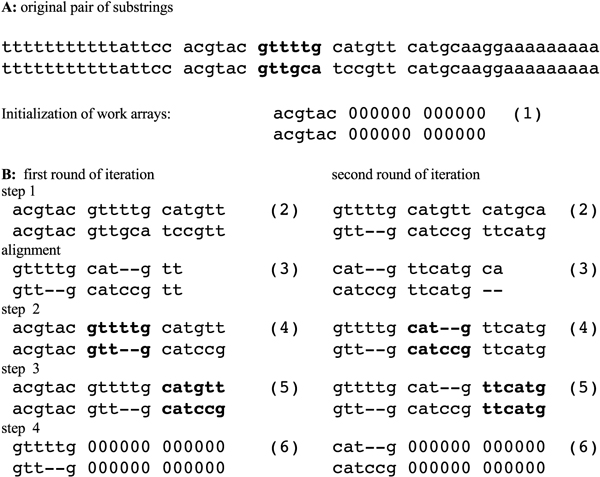
**Match extension by window alignment for W = 6 and K = 2**. Match extension by window alignment for window size *W *= 6 and maximum number of errors *K *= 2. A: We show the original pair of sub-sequences. Beginning from the left, the first window with 3 errors is printed in bold. Both neighbouring windows are separated by blanks. The work arrays (1) are initialized with the contents of the left hand side windows. B: We show the changes of the work arrays for the single steps of iterations described in Section Algorithm. Step 1: The central and right hand side blocks of (1) are overwritten by the next 12 original sequence characters yielding (2). An optimal alignment of these 12 sequence characters is given in (3). Step 2: The central blocks of (2) are overwritten by the first six characters of the alignment (3) and the right hand side blocks of (2) are overwritten by the next 6 original sequence characters yielding (4). Step 3: From the left to the right the first window of size 6 with 3 errors is printed in bold. Its starting position is *T *= 2*W *+1 and the existence of gaps in the central block requires step 4. Step 4: The central blocks of (5) are shifted to the start positions of the work arrays to set the initialization for the next round of iteration shown to the left of the first round. The third round of iteration ends in step 3 without gaps in the central and right hand side blocks of (5) and initiates match extension without gaps as described in the text.

The two work arrays are initialized by substrings of length *W *starting at *x1 *= *e1 *- 2*W *and *x2 *= *e2 *- 2*W*, respectively, and we set *y1 *= *x1 *+ *W *and *y2 *= *x2 *+ *W*. Figure 5 shows a match with three mismatches at the end of the last window of size 6 printed in bold, as well as the resulting initialization of the work arrays. Match extension is now performed taking the following steps of iterations:

1. Overwrite the last 2*W *positions of both work arrays with 2*W *sequence characters beginning in sequence positions *y1 *and *y2*, respectively, and calculate an optimal alignment of these substrings. The length of the alignment may be greater than 2*W *if it contains gaps. An example for *W *= 6 and *K *= 3 is given in Figure 5. It shows under step 1 the contents of both work arrays and the alignment stored.

2. Copy from the aligned sequences the first *W *characters including gap symbols to the work array positions *W *+ 1 to 2*W *and identify the sequence positions of the last copied characters. Then, copy subsequent *W *characters directly from the sequences to completely fill up both work arrays. Hence, part of the alignment is ignored and the last *W *characters of both work arrays are not gap symbols.

3. Count beginning in position *T *= 1 for all sliding windows of size *W *the alignment errors displayed in the two work arrays, stop with the first window that has *K *errors and update *x1*, *y1 *and *x2*, *y2 *to describe the sequence positions of this window. It is the last window in case of Figure 5. For *T *<*W *+ 1 match extension terminates with the result obtained in the previous iteration since match length is not improved.

4. Now *T *is at least *W *+ 1. If the work arrays have gap symbols in positions greater than *T *- *W *or if *T *< 2*W *+ 1, then we move the content of the window beginning in position *T *- *W *to the start positions of the work arrays, update *x1*, *y1 *and *x2*, *y2 *and proceed with step (1). Otherwise a gap-free alignment of length 2*W *starts in position *W *+ 1. We perform match extension without admitting gaps until a window with *K+1 *mismatch errors is found and start all over again.

Obvious modifications apply in case an end-of-sequence symbol occurs in step (1) and terminates match extension.

This heuristic method is fast and was observed to produce near to optimum alignments for highly conserved sequences. The optimal alignment calculated in step (1) uses gap penalty 2 and mismatch penalty 1 in order to minimize internal gaps and does not penalize end gaps. We have not yet optimized the setting of alignment parameters, window size and maximum number of errors per window. Since optimal alignments of short DNA sequences are rarely unique, the result of our match extensions also depends on the particular alignments found. We therefore work on a first improvement of the algorithm that, if possible, studies alternative optimal alignments for a longest possible match extension. Two other ways of improvement are realigning short overlaps of extended matches and joining neighboured matches.

But in spite of considerable potential for improvement our method already provides a close upper bound for the optimal number of errors which is important information if the error count is small and the match is long. Our match extension method cannot lead to a correct optimal alignment by expanding an exact match that is not part of it. We therefore often observe different exact matches leading to the same extended match with different numbers of errors and report the match with the smallest number of errors. However, the gains in alignment quality were observed to be minor compared to match length and the time it takes to generate the same extended match multiple times. We therefore do not extend seed matches which are completely contained in earlier derived extended matches.

### Modes of letter comparison

ClustDB initially searches for clusters of exactly matching substrings of minimal length M using a count sort method that ignores all nucleotide letters other than 'a', 'c', 'g', 't' or the corresponding capital letters. All other letters like 'n' or 'N' are considered to mismatch any nucleotide letter including itself. However, subsequent pairwise match extension with or without gaps uses the full GenBank nucleotide letter alphabet and operates in two modes. By default, nucleotide letters are considered to match if they represent non-disjoint sets of nucleotides (relaxed comparison). While this method of comparison generates nice results in many cases, it also often produces long pairwise matches extending into large runs of n-letters used to represent missing sections of sequences. By switching off relaxed comparison, ClustDB extends pairwise matches considering different letters to mismatch whatever they stand for, a single nucleotide or set of nucleotides. Another option even considers lower case letters to mismatch their corresponding capital letters which allows excluding sequence sections from comparison without loss of sequence information.

### Low complexity sequence problems

Note that our algorithm starts with left maximal clusters of matching substrings. Hence, depending on the minimum match length *M*, a low complexity sequence often generates meaningless large clusters of self-overlapping identical substrings as shown in Table 5. All identical substrings of cluster *CLU *= 1 belong to sequence *SEQ *= 1 and start in equidistant sequence positions *POS*. Optionally, we therefore sort each cluster of matching substrings by their suffix position, check it for runs of close by equidistant positions, and delete such subsets of matches as they are prone to cause large numbers of useless match extensions. In particular, such cases can lead to curious results like reporting one sequence to completely match itself with a small number of gaps.

### Handling of complementary sequences

Many problems like contig assembly require a simultaneous comparison of *N *sequences and their complementary sequences as well. In such a case ClustDB is applied to 2*N *sequences numbered 1,...,*N *for the direct strand sequences and *N *+ 1,...,2*N *for the complementary sequences so that the complement of sequence number *i *receives the number *N *+ *i*. In such a case most left maximal clusters of common substrings have redundant counterparts formed by the corresponding complementary substrings found in complementary sequences. Both clusters collapse to one for self complementary substrings.

Pair wise matches are derived for each pair of sequences in turn. This allows to use a considerable reduction of the maximal number of sequence pairs we need to consider. There are at most *N*(*N *- 1)/2 different pairs of direct strand sequences. Similarly, a pair formed by a direct strand sequence S1 and a complementary sequence S2c produces the same matches as the pair formed by S1c and S2 so that only *N *(*N *- 1)/2 pairs formed by a direct strand sequence and a complementary strand sequence must be considered. Hence, the maximum number of sequence pairs to study is *N*(*N *- 1) and much less than *N*(2*N *- 1), the number of different pairs formed from 2*N *sequences.

### Output of ClustDB

ClustDB outputs different tables depending on the option selected. We can ask for maximally extended exact matches, matches with mismatches, only, and matches with mismatches and gaps. It is also possible to set a lower bound for the length of extended matches. The program package VMATCH first introduced the complete match option. It reduces output to only those matches which prove a complete sequence to be contained in another. We extended this concept to also include matches that prove sequences to overlap. Hence, by our definition, a complete match extends to both sides until the end of at least one sequence. Each line of output starts with a match kind symbol that classifies 6 types of matches.

== sequence 1 matches sequences 2 completely

<< sequence 1 is contained in sequence 2

>> sequence 1 contains sequence 2

-> sequence 1 extends sequence 2 downstream

<- sequence 1 extends sequence 2 upstream

-- incomplete match

If required, ClustDB also stores on disc a list of gap positions for each extended match so that all window alignments are easy to reproduce. Part of a corresponding output is seen in Figure 1. By default, output is reduced to only those lines of the alignments which display errors, since for a typical application of ClustDB, the number of extended matches is large and many matches are very long. Requesting to see all complete alignments is only recommended for repeated runs of the program if there is a clear idea of the amount of output implied.

## Availability of the software

ClustDB is freely available for academic use and can be downloaded from the link given below. The version is provided for the Linux operating system and has been tested on Redhat Linux 9.0. Other versions are available upon request.

Link: 

## Competing interests

The authors declare that they have no competing interests.

## Authors' contributions

JK developed the program ClustDB and drafted the manuscript. FM performed all experiments. BW conceived of the study and participated in its design and coordination. All authors read and approved the final manuscript.

## Figures and Tables

**Table 1:** Histogram of excess errors reported by ClustDB

1 error difference:	1	2	3	4	5	6	7	9	16
2 number of cases:	22	23	5	1	2	1	1	1	1
3 length (kbp):	39	45	43	60	11	36	84	46	106

ClustDB reported non-optimal error counts for 64 (5%) of 1215 alignments. Row one shows the error differences and row three reports the average match length over the number of observed alignments given in row two. The worst case is 16 errors difference for one alignment of length 106 kbp. Figure 1 explains the reason for this difference.

**Table 2:** Runtimes for different applications of ClustDB to 1377 Medicago BACs and their complementary sequences (324 MB of sequence)

*W*	*K*	Runtime	matches
50	5	00:25:05	849
50	10	00:35:35	949
50	15	00:42:57	963
50	20	00:51:32	995
80	5	00:38:31	826
80	10	00:57:29	924
80	15	01:12:40	951
80	20	01:28:31	968
100	5	00:50:45	823
100	10	01:12:37	917
100	15	01:34:00	951
100	20	01:50:46	968
100	30	02:20:48	1015

We present the runtimes in hours (column 3) and the number of complete matches found (column 4) as function of the maximum number of errors (column 2) allowed in all sliding windows of size *W *(column 1). All computations were performed using a Pentium 4 PC with 2 GB of RAM and 2.6 GHz processor speed.

**Table 3:** Twenty pairs of finished Medicago truncatula BACs with obvious sequencing errors detected by ClustDB

type	upstream	downstream	type	upstream	downstream
1	AC136973	AC146774	1	AC151621	AC127168
1	AC143339	AC160629	1	AC153460	AC137837
1	AC174291	AC166286	1	AC170800	AC141922
1	AC174299	AC152886	1	AC147013	AC138010
1	AC148097	AC140551	2	AC135234	AC149208
2	AC138526	AC148775	1	AC144476	AC147431
2	AC125474	AC121246	1	AC174378	AC150647
3	AC142394	AC147435	1	AC148347	AC135229
1	AC138017	AC135800	1	AC140720	AC147000
1	AC135800	AC157757	1	AC147000	AC136142

The two columns named 'type' denote the kind of the problem as described in the section "Testing ClustDB on TIGR's current *Medicago *BAC assembly". The first case is depicted in Figure 2.

**Table 4:** Match quality of overlaps between the BACs shown in Figure 4

BAC1	BAC2	overlap	gaps1	gaps2	errors
AC161749+	AC150776+	19072	4	1	6
AC150776+	AC148343+	21493	0	0	0
AC148343+	AC125368+	10907	0	0	0
AC148343-	AC146751+	1134	0	1	13

Columns 1 and 2 provide BAC identifiers with + for direct strand and - for complement strand, column 3 provides alignment length, and columns 4–6 show gaps and alignment errors including gaps. Hence, the number of aligned nucleotides is given by overlap-gaps1 for BAC1 and overlap-gaps2 for BAC2.

**Table 5:** A typical substring-cluster caused by low complexity sequence

CLU	SEQ	POS	identical substring
1	1	2	t|aaaaaaaaaaaaaaaaaaaa|aaaaat...
1	1	3	ta|aaaaaaaaaaaaaaaaaaaa|aaaat...
1	1	4	taa|aaaaaaaaaaaaaaaaaaaa|aaat...
1	1	5	taaa|aaaaaaaaaaaaaaaaaaaa|aat...
1	1	6	taaaa|aaaaaaaaaaaaaaaaaaaa|at...
1	1	7	taaaaa|aaaaaaaaaaaaaaaaaaaa|t...

The sequence **"taaaaaaaaaaaaaaaaaaaaaaaaat" **generates the left maximal substring-cluster 1 for match length 20. The common substring is formed by a run of 20 letters "a";

## References

[B1] Kent W (2002). BLAT-The BLAST-Like Alignment Tool. Genome Res.

[B2] Sorek R, Safer M (2003). A novel algorithm for computational identification of contaminated EST libraries. Nucleic Acids Research.

[B3] Kurtz S, Choudhuri J, E O, Schleiermacher C, Stoye GRJ (2001). REPuter: the manifold application of repeat analysis on a genomic scale. Nucleic Acids Research.

[B4] Delcher A, Philippy A, J C, SL S (2002). Fast algorithms for large scale genome alignment and comparison. Nucleic Acids Research.

[B5] Kurtz S, Schleiermacher C (1999). REPuter: fast computation of maximal repeats in complete genomes. Bioinformatics.

[B6] Abouelhoda M, S K, Ohlebusch E (2004). Replacing Suffix Trees with Enhanced Suffix Arrays. Journal of Discrete Algorithms.

[B7] Burkhardt S, Crauser A, Farragina P, Lenhof H, Vingron M (1999). q-gram based searching using a suffix array (quasar). Int Conf RECOMB, Lyon, April 1999.

[B8] Höhl M, Kurtz S, Ohlebush E (2002). Efficient multiple genome alignment. Bioinformatics.

[B9] Lefebvre A, Lecroq T, Dauchel H, Alexandre J (2003). FORRepeats: detects repeats on entire chromosoms and between genomes. Bioinformatics.

[B10] Kleffe J, Möller F, Wittig B (2006). ClustDB: A high-performance tool for large scale sequence matching. Proceedings of DEXA, Krakau, Sep 4–8, 2006.

[B11] Warburton PE, Giordano J, Cheung F, Gelfand Y, Benson G (2004). Inverted repeat structure of the human genome: the X-chromosome contains a preponderance of large, highly homologous inverted repeats that contain testes genes. Genome Res.

[B12] Mielordt S, Grosse I, Kleffe J (2006). Data structures for genome annotation, alternative splicing, and validation. Lecture Notes in Computer Science – Proceedings of DILS06, Hinxton July 20–22, 2006.

[B13] Hiller M, Huse K, Szafranski K, Jahn N, Hampe J, Schreiber S, Backofen R, Platzer M (2004). Widespread occurrence of alternative splicing at NAGNAG acceptors contributes to proteom plasticity. Nature Genetics.

[B14] Check E (2006). Mix and match: the hunt for what makes us human. Nature.

[B15] Hamborg T, Kleffe J (2006). MPI-ClustDB: A fast String Matching Strategy Utilizing Parallel Computing. Lecture Notes in Informatics – Proceedings of GCB 2006, Tuebingen, September 20–22, 2006.

